# Glutamine synthetase gene *PpGS1.1* negatively regulates the powdery mildew resistance in Kentucky bluegrass

**DOI:** 10.1093/hr/uhac196

**Published:** 2022-08-30

**Authors:** Xiaoyang Sun, Fuchun Xie, Yajun Chen, Zhixin Guo, Lili Dong, Ligang Qin, Zhenjie Shi, Liangbing Xiong, Runli Yuan, Wenjing Deng, Yiwei Jiang

**Affiliations:** College of Animal Science and Technology, Northeast Agricultural University, Harbin, China; College of Animal Science and Technology, Northeast Agricultural University, Harbin, China; College of Animal Science and Technology, Northeast Agricultural University, Harbin, China; College of Horticulture, Northeast Agricultural University, Harbin, China; College of Horticulture, Northeast Agricultural University, Harbin, China; College of Horticulture, Northeast Agricultural University, Harbin, China; College of Animal Science and Technology, Northeast Agricultural University, Harbin, China; College of Horticulture, Northeast Agricultural University, Harbin, China; College of Horticulture, Nanjing Agricultural University, Nanjing, China; College of Horticulture, Northeast Agricultural University, Harbin, China; College of Horticulture, Northeast Agricultural University, Harbin, China; College of Horticulture, Northeast Agricultural University, Harbin, China; Department of Agronomy, Purdue University, West Lafayette, IN, USA

## Abstract

Excessive nitrogen (N) application may induce powdery mildew (PM) in perennial grasses, but the resistance mechanisms to PM remain unclear. This study evaluated the physiological and molecular mechanisms of PM resistance affected by N supplies in Kentucky bluegrass (*Poa pratensis* L.). Cultivar ‘Bluemoon’ (N tolerant) and ‘Balin’ (N sensitive) were treated with low N (0.5 mM), normal N (15 mM), and high N (30 mM) for 21 d in a greenhouse. With increasing N levels, the disease growth was more severe in ‘Balin’ than in ‘Bluemoon’. RNA-seq and weighted gene coexpression network analysis revealed that the *PpGS1.1* gene encoding glutamine synthetase was a potential hub gene for PM resistance after comparisons across cultivars and N treatments. The N metabolism pathway was connected with the plant–pathogen interaction pathway via *PpGS1.1*. The expression of *PpGS1.1* in rice protoplasts indicated that the protein was located in the nucleus and cytoplasm. Overexpression of *PpGS1.1* in wild-type Kentucky bluegrass increased carbon and N contents, and the transgenic plants became more susceptible to PM with a lower wax density. The most differentially expressed genes (DEGs) for N metabolism were upregulated and DEGs for fatty acid metabolism pathway were downregulated in the overexpression lines. The results elucidated mechanisms of PM resistance in relation to N metabolism in Kentucky bluegrass.

## Introduction

Kentucky bluegrass (*Poa pratensis* L.) is a popular turfgrass grown in temperate regions [1]. However, powdery mildew [*Blumeria graminis* (DC.) Speer] (PM) is prone to occur in Kentucky bluegrass and other turfgrasses when cultivated in low airflow, high relative humidity, and shaded areas [2]. Although some Kentucky bluegrass ecotypes with higher PM are found in wet meadows, none of the ecotypes shows complete resistance [3, 4]. Powdery mildew mainly infects stems and leaves from seedlings to mature turf. The infected leaves are covered with white mycelium and then turn yellow, and subsequently can die from disease infection. A previous study revealed that *B. graminis*, one of turfgrass obligate parasitic PM species, mainly infected turfgrass species such as creeping bentgrass (*Agrostis stolonifera* L.), Kentucky bluegrass, and bermudagrass (*Cynodon dactylon* L.) [2]. Recently, the rDNA sequence of *B. graminis* was reported in Kentucky bluegrass, providing a reference for further studying PM in turfgrasses [2].

At the molecular level, infection by PM leads to changes of gene expression in plants. In wheat (*Triticum aestivum* L.), PM is caused by the fungus *B. graminis*, the same fungus species found in Kentucky bluegrass [2, 5]. Most of the known PM resistant genes in wheat are qualitative resistance (R) genes, and their functions can be rapidly lost due to the variations in PM races [6]. Thus, plants need a stronger immune system against PM. Two major divisions of the plant immune systems have been identified: effector-triggered immunity (ETI) and pathogen-associated molecular patterns-triggered immunity (PTI) [7]. PTI improved PM resistance in *Arabidopsis thaliana* and barley (*Hordeum spontaneum* L.) [8]. Likewise, calcium-dependent protein kinases in PTI enhanced PM resistance in grape (*Vitis vinifera* L.) and *Arabidopsis* by interacting with potentially phosphorylate mitogen-activated protein kinases [9]. In ETI, the most-characterized plant R proteins are in the category of the nucleotide binding site-leucine-rich repeat (NB-LRR) protein superfamily [7]. Several isolated R genes for PM belong to this class, including the *R* genes of barley [10] and *Resistance to Powdery Mildew* gene*s* (*RPW*) of *Arabidopsis* [11]. In addition, ectopic expressions of *R* in *Arabidopsis* and rice (*Oryza sativa* L.) boosted PTI signaling by increasing H_2_O_2_ production [12]. However, our knowledge on molecular mechanisms of PM is still limited, especially in perennial grasses.

The PM invasion can be protected by a waxy cuticle layer atop the cell wall [13]. The *rst1* (*RESURRECTION1*) mutant enhanced susceptibility to PM with significantly increased funicular waxes on leaves in *Arabidopsis* [14]. However, the removal of total leaf cuticular waxes caused a 20% decrease in germination of *B. graminis* conidial and differentiation in barley [15]. Fatty acids are major substrates for wax biosynthesis, playing a role in resistance to *Erysiphe graminis* in barley and *Magnaporthe grisea* in rice [16, 17]. Fatty acids related genes have been characterized in plants that participate in disease resistance including *Arabidopsis* and oilseed rape (*Brassica napus* L.) [18, 19]. The hypersensitive cell death response can be activated by a MYB transcription factor that regulates fatty acids biosynthesis in *Arabidopsis* [18]. In Kentucky bluegrass, overexpression of *PpCER1–2* encoding ECERIFERUM related wax biosynthesis affected fatty acids contents [20]. Although the penetration of fungus through the wax layer is crucial for plant-fungus interactions, a successful colonization largely depends on the capacity of the pathogen to obtain the nutrients from the host. As a macronutrient, N rapidly stimulates nutrient transport and assimilation as well as carbon/nitrogen metabolism [21]. Fatty acids are carbon source for plants to feed fungi, and easier colonization can be often achieved with adequate and enough N in plants [22]. A previously study showed that low N significantly increased fatty acids content in *Aspergillus oryzae* [23]. More interestingly, increasing the amount of nitrogenous compounds decreased fatty acid contents in winter mustard (*Brassica juncea* L.) and oilseed rape [24, 25]. However, how N affects the fatty acid content remains unclear in perennial grasses.

Adequate N supply is a key factor for plant resistance to pathogens [26, 27]. Excessive N application stimulates plant growth, thus promoting disease occurrence in grain crops and vegetables [26, 28]. Several fungal pathogenicity can be controlled by N starvation in tomato (*Solanum lycopersicum* L.) [26]. Furthermore, transcriptional patterns N metabolism genes were induced in response to pathogen inoculations [26, 29–31]. The susceptibility to *Pseudomonas syringae* and *Ralstonia solanacearum* decreased by silencing N metabolism genes including nitrate reduction (*NR*), nitrite reductase (*NiR*) and glutamate synthase (*GOGAT*), but increased in *NiR1*-overexpressed tomato plants [26]. Previous studies showed that the glutamine synthetase (*GS*) gene was mainly upregulated in rust infected coffee (*Coffea arabica* L.) leaves. Moreover, *GS* expression was positively correlated with the patterns of defense marker genes such as *PAL3* and *CHS* in the fungus-infected bean (*Phaseolus vulgaris* L.) leaves [29]. In tomato (*Lycopersicon esculentum* Miller.), cytosolic *GS* was considered a defense-related protein during pathogen stress [29]. Interestingly, *GS* can form a complex with asparagine synthetase (*AS*) or glutamate dehydrogenase (*GDH*) for plant defense responses during plant–pathogen interaction [30, 31]. Nevertheless, detailed research is lacking on the molecular regulatory mechanisms in perennial grass species infected by PM.

The objective of this study was to explore the molecular mechanisms of PM infection of Kentucky bluegrass in response to N supplies. We examined morphology, disease spot growth, and N assimilation enzyme activities and antioxidant metabolism under different N levels in two cultivars (‘Balin’ and ‘Bluemoon’) with contrasting N responses. Furthermore, we identified DEGs in response to N application mediated by PM. We identified a hub gene responding to PM that revealed the defensive mechanisms through genetic transformation, microstructure, and related genes expression level. The current work would provide a good basis for genetic improvement of pathogen resistance and N utilization in Kentucky bluegrass and other related perennial grass species.

## Results

### Powdery mildew severity degree under three N concentration levels

**Figure 1:**
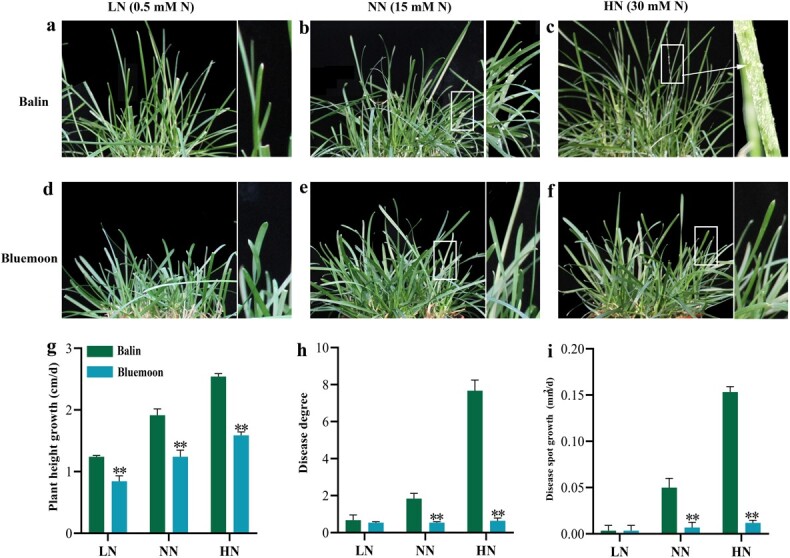
Response of two Kentucky bluegrass cultivars to powdery mildew. **a**–**f** Images of the cultivars ‘Bluemoon’ and ‘Balin’ in response to powdery mildew under nitrogen (N) treatments. The white boxes represent locally enlarged uninfected and infected leaves. **g**–**i** Plant height growth, disease degree and disease spot growth in two cultivars. The bars represent standard deviation. Asterisks indicate a significant difference to ‘Balin’ (^**^P < 0.01, ^*^P < 0.05). LN, NN, and NH represent low, normal, and high N, respectively. Green bar indicates ‘Balin’ and blue bar indicates ‘Bluemoon’.

The plant heights of ‘Bluemoon’ were 32.0%, 34.7%, and 36.4% lower than that in ‘Balin’ under LN, NN, and HN, respectively (Fig. 1a–g). With increasing N level supplies, no obvious PM symptoms appeared on leaves in ‘Bluemoon’, while the circular and irregular white powdery fungal colonies covered the entire leaf surface in ‘Balin’ (Fig. 1a–f). Disease severity degree and disease spot growth were maintained at a stable level in ‘Bluemoon’, but were aggravated in ‘Balin’ (Fig. 1h–i). Under LN conditions, disease severity degree and disease spot growth were not significantly different between the two cultivars (Fig. 1h–i), but disease degree of ‘Bluemoon’ was 71.0% and 91.8% lower than in ‘Balin’ under NN and HN, respectively (Fig. 1h). Similar trends in disease spot growth were found under NN and HN treatments, with 88.0% and 92.8% lower in ‘Bluemoon’ than in ‘Balin’, respectively (Fig. 1i).

### Nitrogen assimilation enzyme activities and antioxidant metabolism

**Figure 2:**
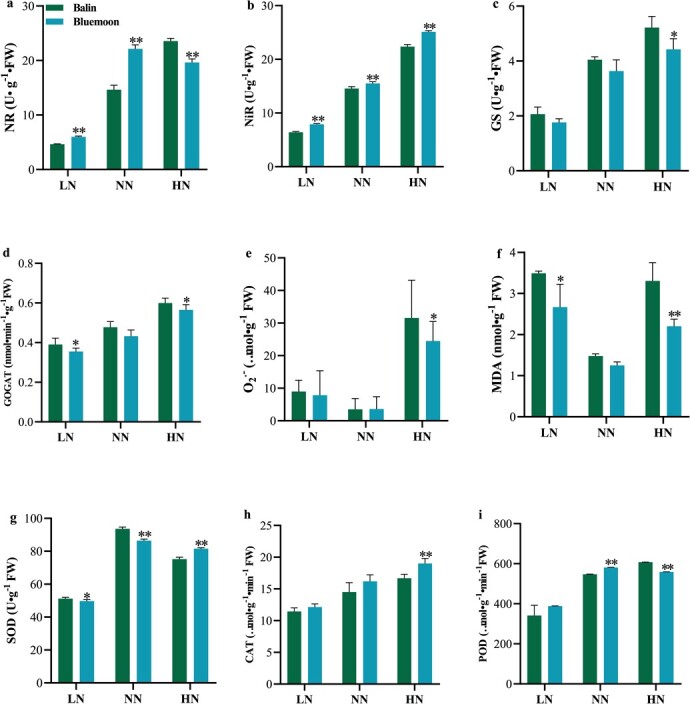
Nitrogen related-metabolism enzyme activities and antioxidant metabolism. **a**–**i** Nitrate reductase (NR), nitrite reductase (NiR), glutamine synthetase (GS), glutamate synthase (GOGAT), superoxide dismutase (SOD), catalase (CAT), and peroxidases (POD) activity; superoxide anion (O_2_^•-^) and malondialdehyde (MDA) content in ‘Balin’ and ‘Bluemoon’ of Kentucky bluegrass under different nitrogen (N) treatments. The bars represent standard deviation. Asterisks indicate a significant difference to ‘Balin’ (^**^P < 0.01, ^*^P < 0.05). LN, NN, and NH represent low, normal, and high N, respectively. Green bar indicates ‘Balin’ and blue bar indicates ‘Bluemoon’.

Compared to ‘Balin’, ‘Bluemoon’ exhibited a significant increase of NR activity by 29.0% under LN and 51.1% under NN (Fig. 2a). NiR activities in ‘Bluemoon’ was markedly increased under LN (22.8%) and HN (12.3%) compared to that in ‘Balin’ (Fig. 2b). By contrast, GS activity was lower in ‘Bluemoon’ under LN and HN levels (Fig. 2c). Furthermore, GOGAT activity increased under NN and HN treatments, while GOGAT activity in ‘Bluemoon’ was still 9.4% and 5.8% lower relative to ‘Balin’, respectively (Fig. 2d). Overall, two cultivars showed similar trends in N assimilation activities with increasing N supplies.

The O_2_^•-^ and MDA contents were markedly increased in the two cultivars in response to LN and HN (Fig. 2e and f). Especially at the HN level, the O_2_^•-^ content in ‘Balin’ was nearly 1.3-fold higher than that in ‘Bluemoon’ (Fig. 2e). Meanwhile, MDA content also increased by 30.8% and 50.2% more in ‘Balin’ compared to ‘Bluemoon’ under LN and HN (Fig. 2f). Antioxidant enzyme activities did not differ in two cultivars under LN (Fig. 2g–i). Under the HN level, POD activity was 8.0% lower and SOD activity was 8.5% higher in ‘Bluemoon’ compared to ‘Balin’ (Fig. 2g and i). For NN and HN treatments, CAT activity was 11.7%, and 13.9% higher in ‘Bleumoon’ than that in ‘Balin’, respectively (Fig. 2h). This suggested that ‘Bluemoon’ maintained a stronger capacity in scavenging O_2_^•-^ to reduce oxidative damage.

Pairwise correlation coefficients among all the traits are presented in Table S5 (see online supplementary material). Across two cultivars and three N levels, 29 significant correlations were identified among the traits. Specifically, O_2_^•-^ was positively correlated with MDA (*r* = 0.45), CAT activity (*r* = 0.48), and N assimilation enzymes (*r* = 0.43 to 0.77). MDA had a positive correlation with SOD activity (*r* = 0.72) and negative correlation with POD activity (*r* = −0.47), but no correlations were found between MDA and N assimilation enzymes. Antioxidant enzymes all had positive correlations with N assimilation enzymes.

### Genes expression and pathway enrichment

PCA analysis on transcriptome data indicated that six groups were well separated in response to N rates (Fig. S2a, see online supplementary material). We used a hierarchical clustering method to examine the overall expression trend of the DEGs (Fig. S2b, see online supplementary material). Differences in expression levels were observed in most of the DEGs under different N treatments. To find key genes, DEGs were compared between two cultivars (‘Balin’ vs ‘Bluemoon’), and a total of 69 281 were discovered at HN, 36 432 at NN, and 45 671 at LN, respectively (Fig. S3a, see online supplementary material). Notably, KEGG enrichment analyses showed that ‘plant–pathogen interaction’ was the most significantly enriched in the three comparisons (*P_NN_* = 1.6E-04, *P_HN_* = 0.02, *P_LN_* = 4.4E-08) (Fig. S3b, see online supplementary material).

### Identification of gene co-expression modules and hub gene

**Figure 3:**
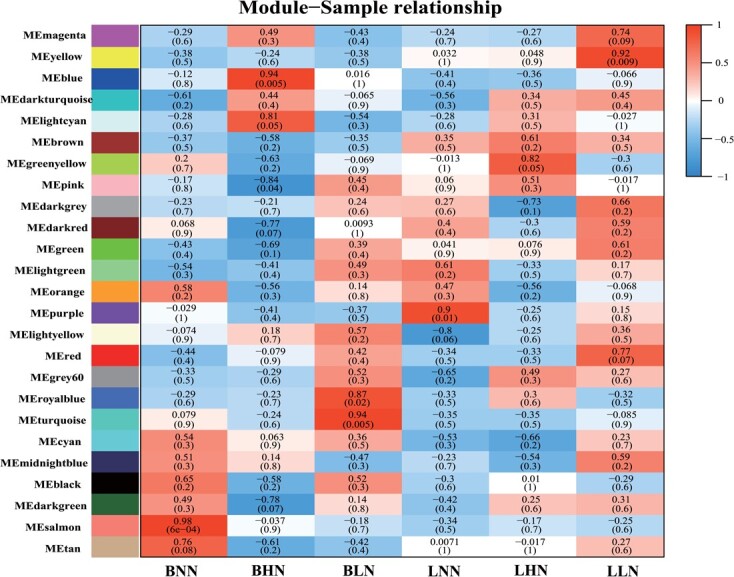
Modules relationship patterns of transcripts in two cultivars of Kentucky bluegrass via WGCNA. The abscissa represents the sample, and the ordinate represents the module. The number in each grid indicates the correlation between the module and the sample: the closer to 1, the stronger positive correlation; the closer to −1, the stronger negative correlation. Each cell consists of the number of corresponding correlations and *P*-values (in parentheses). (BLN, BNN, BHN = ‘Balin’ under low, normal, and high nitrogen treatments, respectively. LLN, LNN, LHN = ‘Bluemoon’ under low, normal, and high nitrogen treatments, respectively.)

The co-expression modules via module-sample relationship were analysed in two cultivars under different N levels. There were 43 333 genes selected from a total of 522 590 unigenes, and then subjected to WGCNA algorithm. Twenty-five gene co-expression modules related to all treatments were ultimately detected (Fig. 3), with the number of genes ranging from 151 to 6661 in these modules.

**Figure 4:**
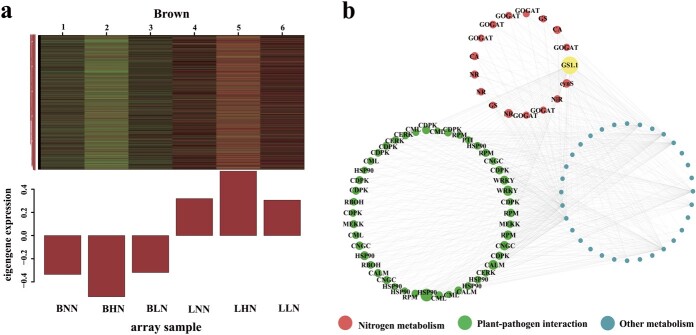
Visual analysis of co-expression correlation of genes in brown module. **a** Co-expressed genes in brown module are shown in bar graphs. **b** Visual analysis of co-expression correlation of genes in brown module via Cytoscape. Each node represents a DEG, and the connecting lines (edges) between genes represent co-expression correlation. The size of circles is proportional to connectivity. The red, green, and blue circles represent gene in ‘nitrogen metabolism’, ‘plant–pathogen interaction’, and other metabolism pathways, respectively. (BLN, BNN, BHN = ‘Balin’ under low, normal, and high nitrogen treatments, respectively. LLN, LNN, LHN = ‘Bluemoon’ under low, normal, and high nitrogen treatments, respectively.)

The trend of eigengenes (also called hub/key genes) in most modules (e.g. yellow, cyan, and salmon modules) was nearly irregulated in the two cultivars (Fig. 3). It is noteworthy that the genes in the brown module, which showed the opposite expression pattern, were highly expressed in ‘Bluemoon’ but lower expressed in ‘Balin’ (Fig. 4a). Further, the greenyellow module had a significant positive correlation with ‘Bluemoon’ but a negative correlation with ‘Balin’ under HN stress (Fig. S4, see online supplementary material). A total of 5398 genes were found in the brown module and 1027 genes in the greenyellow module. Taken together, these results indicated that the genes in brown and greenyellow modules played an important role in resisting PM of Kentucky bluegrass.

The software Cytoscape was used to visualize the hub genes in the brown and greenyellow modules. As a hub gene, *GS1.1* (TRINITY_DN134338_c2_g1) had co-expression correlations with ‘nitrogen metabolism’ (e.g. *NR*, *GOGAT*, and *CA* genes) and ‘plant–pathogen interaction’ (e.g. *CDPK*, *RBOH*, *WRKY* genes) pathways (Fig. 4b). Then, after screening in the greenyellow module, a total of 21 genes were interconnected to form a complex regulatory network including genes such as *NR*, *GS*, *GOGAT*, *HSP90* and *CDPK* (Fig. S4, see online supplementary material). The results revealed that genes related to the ‘nitrogen metabolism’ and ‘plant–pathogen interaction’ pathways were strongly related to each other (Fig. 4, Fig. S4, see online supplementary material). The hub gene *GS1.1* in the brown module suggested a previously unknown function, indicating that it could be a potential gene for further research.

### DEGs in the plant–pathogen interaction and nitrogen metabolism pathway

**Figure 5:**
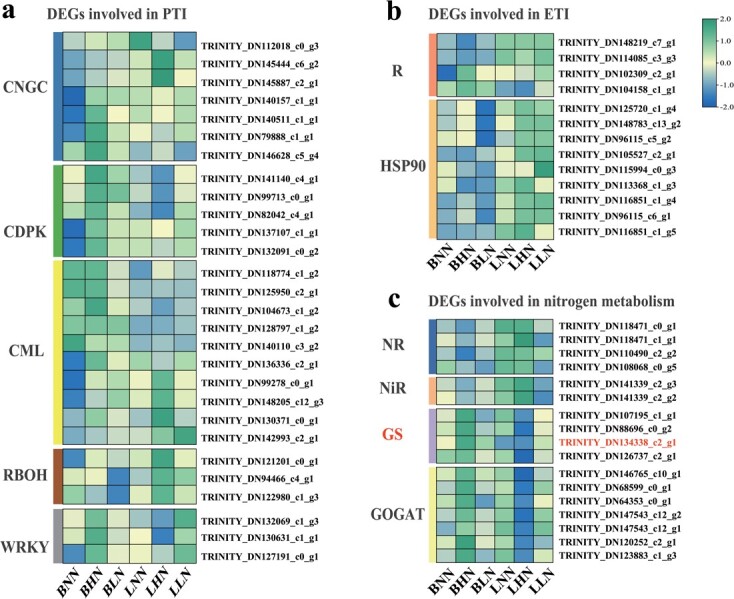
Analysis of differentially expressed genes (DEGs) of two cultivars in response to powdery mildew. **a**–**c**. Heatmaps of DEGs in PTI, ETI, and nitrogen metabolism, respectively. The bar indicates the scale of the expression levels for each gene (FPKM) under the different treatments. The color represents the expression levels of genes. (BLN, BNN, BHN = ‘Balin’ under low, normal, and high N treatments, respectively. LLN, LNN, LHN = ‘Bluemoon’ under low, normal, and high N treatments, respectively.)

KEGG analysis showed that the proportions of DEGs from each pathway were from 2.9% to 33.3% in the brown module (Fig. S5, see online supplementary material). The proportions of ‘nitrogen metabolism’ and ‘plant–pathogen interaction’ pathway were 2.9% and 8.6%, respectively (Fig. S5, see online supplementary material). Next, DEGs were analysed in the brown module enriched in the ‘plant–pathogen interaction’ pathway. The majority of DEGs were involved in the regulation of Ca^2+^ signal transduction. The *CNGC* encoding cyclic nucleotide gated channel, *CML* encoding calmodulin and *CDPK* genes mediating signal transmission were highly expressed in ‘Balin’ under HN, but the expression of these genes was relatively complex in ‘Bluemoon’ (Fig. 5a). The *Rboh* genes related to ROS bursts had the highest expression level in ‘Bluemoon’ under the HN treatment (Fig. 5a). Furthermore, the *WRKY2* and *WRKY26* transcription factors had higher expression levels in ‘Bluemoon’ compared to ‘Balin’ under HN, and were highly expressed in ‘Bluemoon’ under LN (Fig. 5a). The *HSP* genes involved in a subsequent ETI immunization process were specifically and highly expressed in ‘Bluemoon’ (Fig. 5b). Among the *R* genes that mediate R protein activity, two *RGA* genes were strongly expressed in ‘Bluemoon’ compared to ‘Balin’ under three N levels. The *RPM* and *RPP* genes were highly expressed in ‘Balin’ under the HN treatment (Fig. 5b).

Furthermore, DEGs in the ‘nitrogen metabolism’ pathway were examined in the brown module. These DEGs involved in the process of N absorption and transformation were highly expressed in ‘Bluemoon’ under NN and HN treatments (Fig. 5c). Especially at HN, the upregulation of *NR* and *NiR* expressions indicated the enhanced ability of plants converting NO_3_^−^ to NH_4_^+^ (Fig. 5c). However, the highly expressed *GS* and *GOGAT* genes in ‘Balin’ might promote the assimilation of NH_4_^+^ to generate more glutamate in ‘Balin’, relative to ‘Bluemoon’ (Fig. 5c).

### Bioinformatics analysis and subcellular localization of *PpGS1.1*

**Figure 6:**
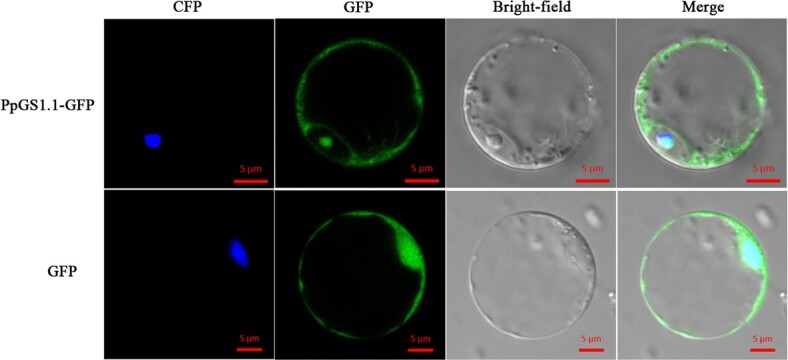
Colocalization of PpGS1.1-GFP and nuclear marker OsGhd7-CFP in rice protoplasts. The CFP fluorescence is blue and the GFP is green. Merge is created by merging the GFP and OsGhd7-CFP fluorescence images. Scale bars 5 μm.

The *PpGS1.1* gene has a 1104 bp ORF and two domains (Gln-synt N and Gln-synt C), which encode a protein with 368 amino acids (Fig. S6a, see online supplementary material). A phylogenetic tree analysis revealed that the *GS1.1* of *P. pratensis* and *Brachypodium distachyon* shared 99.7% similarity (Fig. S6b, see online supplementary material). To determine the subcellular localization of PpGS1.1, the fusion constructs of GFP and a PpGS1.1-GFP were introduced into rice protoplasts with the nuclear marker OsGhd7-CFP. Under a laser confocal microscope, the fluorescence of PpGS1.1 coincided with the fluorescence of a nuclear marker located in the nucleus (Fig. 6). There was a strong fluorescence of GFP in the cytoplasm, suggesting that this gene was also expressed in the cytoplasm (Fig. 6).

### Overexpression of *PpGS1.1* on powdery mildew resistance

**Figure 7:**
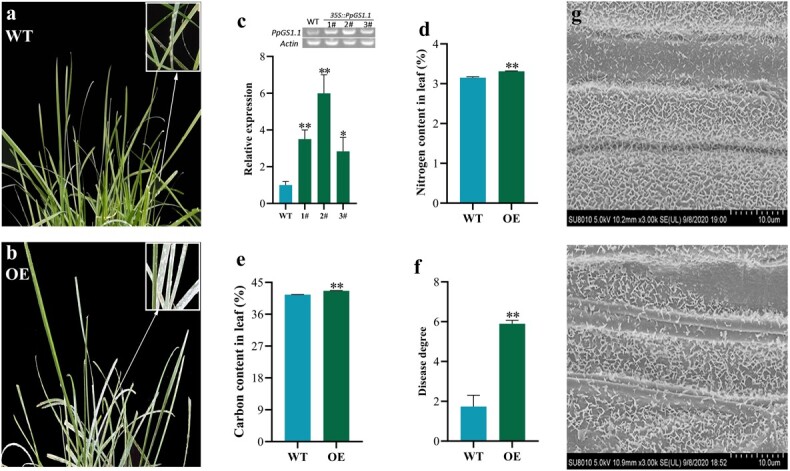
Identification of *PpGS1.1*-overexpressing lines with contrasting wild type (WT). **a**, **b** Phenotypes of the overexpression lines (OE) and WT plants. **c***PpGS1.1* gene relative expression in OE and WT and electrophoresis detection is in the upper right corner. **d**, **e** Leaf nitrogen and carbon content. **f** Disease degree. **g, h** SEM observation. The bars represent standard deviation. Asterisks indicate a significant difference to WT (^**^P < 0.01, ^*^P < 0.05).

To determine the function of the *PpGS1.1* gene, we constructed a *PpGS1.1* overexpression vector driven by the CaMV 35S promoter and introduced it into wild-type plants (Fig. 7a and b). The expression level of *PpGS1.1* significantly increased in the transformed lines according to electrophoresis detection and qPCR assays (Fig. 7c). The carbon and N contents in transgenic plants increased by 3.1% and 2.6%, respectively, relative to that in WT (Fig. 7d and e). Moreover, the inoculation assays demonstrated that transgenic lines were more susceptible to PM, with a disease index significantly higher (1.9-fold) than that in WT (Fig. 7a, b and f). Interestingly, SEM results showed a reduction of wax density in overexpressing *PpGS1.1* lines, compared to that in the WT (Fig. 7g and h).

### Comparative transcriptome profiling between *PpGS1.1*-overexpression lines (OE) and wild-type (WT)

To identify the regulatory genes in *PpGS1.1*-overexpression lines, we performed transcriptome sequencing of OE and its background WT. A total of 7862 DEGs were identified, including 3049 upregulated and 4813 downregulated genes in OE compared to WT (Fig. S7a, see online supplementary material). These DEGs were enriched in the pathways of‘nitrogen metabolism’ (ko00910; *P*-value = 1.7e-04) and ‘glyoxylate and dicarboxylate metabolism’ (ko00630; *P*-value = 7.1e-04) inOE (Fig. S7b, see online supplementary material). Furthermore, GO enrichment patterns were generally consistent with KEGG analysis. The pathway ‘oxidation–reduction process’ in the biological process category (GO:0055114; *P*-value = 3.7e-12) was significantly enriched in OE compared to WT (Table S6, see online supplementary material). In the molecular function category, ‘ADP binding’ (GO:0043531; *P*-value = 9.3e-14) and ‘oxidoreductase activity’ (GO:0016491; *P*-value =1.6e-11) were the most abundant pathways (Table S6, see online supplementary material).

### Candidate genes between OE and WT

**Figure 8:**
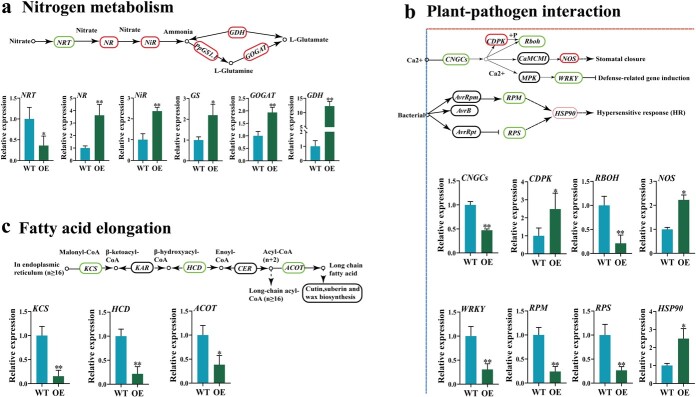
Analysis of differentially expressed genes (DEGs) between wild type (WT) and *PpGS1.1*-overexpression (OE) lines based on transcriptome sequencing. **a** DEGs in nitrogen metabolism pathway. **b** DEGs in plant pathogen interaction pathway. **c** DEGs in fatty acid elongation pathway. The bars represent standard deviation. Asterisks indicate a significant difference to WT (^**^P < 0.01, ^*^P < 0.05).

Genes involved in N transport and assimilation were differentially expressed in OE compared to WT. Expressions of *NR*, *NiR*, *GS*, *GOGAT* were upregulated (~2.0-fold) in transgenic lines (Fig. 8a). Remarkably, *GDH* encoding glutamate dehydrogenase in OE was significantly higher (12.3-fold) than that in WT. However, the *NRT* gene was downregulated in transgenic lines to about half of that in WT (Fig. 8a).

Genes related to plant–pathogen interaction induced by overexpression of the *PpGS1.1* gene were differentially expressed in transgenic lines. R genes, like *RBOH*, *RPM* and *RPS* were downregulated (~ 0.5-fold) in OE lines compared to the WT (Fig. 8b). *CNGCs* encoding cyclic nucleotide gated channel and *WRKY* encoding a type transcription factor all exhibited similar patterns to R genes (Fig. 8b). Compared to WT, *CDPK* encoding calcium-dependent protein kinase, *NOS* encoding nitric-oxide synthase and *HSP90* encoding heat shock protein were all highly expressed in OE lines (Fig. 8b).

In general, all DGEs in ‘fatty acid elongation’ of this pathway were downregulated in OE plants. Specifically, *KCS* encoding β-keto acyl-coA synthase condensing enzyme, *HCD* encoding hydroxyacyl-CoA dehydratase and *ACOT* encoding acyl-coenzyme A thioesterase were all higher in OE than those in WT (Fig. 8c). Overexpression of *PpGS1.1* induced upregulation of genes related to N metabolism, but candidate genes in the fatty acid elongation pathway and *R* genes in the plant pathogen interaction pathway were downregulated (Fig. 8).

## Discussion

Nitrogen status strongly affects plant growth and adaptation to environmental stresses in Kentucky bluegrass [1, 32]. A better understanding of the mechanisms of N-mediated plant response to pathogens is crucial for proper disease management. However, N-affected PM disease degree is still unclear in Kentucky bluegrass. In this study, we identified candidate genes that responded to *B. graminis* infection at different N concentrations after inoculation in both N-sensitive ‘Balin’ and N-tolerate ‘Bluemoon’ cultivars. By WGCNA analysis and overexpression of key N-metabolism gene we elucidated a relationship between N metabolism and plant immunity to pathogens, and demonstrated that the *GS* negatively regulated the PM resistance in Kentucky bluegrass.

As N supply is sufficient, the GS/GOGAT cycle activates and promotes N assimilation into glutamate rather than asparagine synthetase metabolism to participate in plant growth [29–31]. ‘Balin’ assimilated more N for plant growth through the higher N assimilation activities, especially under HN (Fig. 2a–d). This indicated that the application of HN might make *GS* difficult to form a complex with *AS* to respond to pathogen invasion in the N-sensitive cultivars. Therefore, the spread of lesions had nearly tripled in the N-sensitive cultivar ‘Balin’ than that in the N-tolerant ‘Bluemoon’ under HN, indicating that PM induced by N varied among the cultivars. Under HN treatment, other high-N-content cultivars also showed aggravation of powdery mildew compared with low-N-content cultivars (Fig S1a, see online supplementary material). Moreover, increasing GS can regulate the expression level of MDA and membrane peroxidation [33]. Antioxidant enzymes play a pivotal role in reducing the potential oxidative damage, especially under N stress in Kentucky bluegrass [32, 34]. Higher antioxidant activities in ‘Bluemoon’ compared to ‘Balin’ were beneficial to maintaining the integrity of cell membranes under HN (Fig. 2g and h). The positive correlations between partial N metabolism and antioxidant enzyme activities confirmed that changes in N metabolism enzymes affected the antioxidant systems in Kentucky bluegrass (Table S5, see online supplementary material).

Previous studies showed that cytoplasmic *GS* played a key role in regulating N starvation stress in Kentucky bluegrass [32] and against several diseases in other plant species [29–31]. Overexpression of *GS* increased N content in sorghum (*Sorghum bicolor* L.) and rice. After LN treatment, the expression of *GS1.1* was up-regulated (Fig. 5c), which was consistent with the results found in rice seedlings [35]. Our results also demonstrated that overexpression of *PpGS1.1* lines had higher carbon and N contents under NN supply (Fig. 7d and e), and overexpressing of *PpGS1.1* made plants less immune to PM (Fig. 7a, b and f). Here, we proved that *GS* might affect the onset of PM by increasing the N content in the plants. Overexpression of *GS1.1* gene improved the nitrogen-use efficiency of Kentucky bluegrass, and aggravated the occurrence of PM disease. Future breeding work could be promoted by selecting candidate genes that interact with *GS1.1* [36].

Previous reports indicate that glutamate (Gln) could act as damage-associated molecular pattern (DAMPs), causing systemic changes in Ca^2+^ concentration, and thus inducing the spread of defense signals throughout the PTI [37]. The overexpression of the *CDPKs* gene positively regulates phytohormonal signals and improves resistance to PM [9, 38]. In addition, CDPKs can directly bind the *RbOH* gene to regulate plant C and N metabolism by inhibiting or activating NR enzyme [21, 38]. In this study, the *PpGS1.1* gene had a co-expression correlation between ‘nitrogen metabolism’ and ‘plant–pathogen interaction’ pathways (Figs. 3 and 4). In *PpGS1.1* overexpression lines, *CDPK* and *NR* were upregulated compared with WT (Fig. 8a and b). However, the WGCNA results showed that *CDPK* and *NR* expressed in the opposite pattern in two cultivars under HN (Fig 5a and c). The susceptibility to *P. syringae* and *R. solanacearum* disease was reduced by silencing N metabolism *NR* genes in tomato [26]. Whether *CDPK* inhibits or promotes *NR* is unclear, which needs further research in Kentucky bluegrass (Fig. 8a and b)*.* We speculated that the upregulation of the *PpGS1.1* gene enabled most of Gln to inhibit the PTI. The results supported that *GS* may not only form a complex with *GDH* or *AS* but also forms a gene complex with *CDPK* and *NR* for regulating plant resistance to PM [30, 31].

Pathogens suppress or block the PTI response, and the plants correspondingly activate the ETI response [7]. As a negative regulator, the decreased expression of the RIN protein enhanced *RPM* gene-mediated PM resistance [10, 12]. Our results indicated that the higher expression level of the *RIN* gene in ‘Balin’ decreased its PM resistance under HN (Fig. 5b). The upregulated expression of *RPM* genes in ‘Bluemoon’ treated with HN enhanced plant resistance by activating *HSP90* (Fig. 5b). Therefore, *RPM* could be a key gene in the response to PM in Kentucky bluegrass, similar to the role of *RPM8* in PM resistance in *Arabidopsis* [12]. Also in *Arabidopsis*, the interaction between *HSP90* and *RAR* identified the effector secreted by pathogens [39]. It appeared that *HSP90* genes identified in the brown module played a positive role in disease resistance by inhibiting the spread of pathogenic substances in ‘Bluemoon’ (Fig. 5b).

Plants are protected from PM infection by a waxy cuticle layer atop the cell wall [13]. It was surprising that the wax density on the plant surface was significantly reduced in the overexpression lines (Fig. 7g and h). Fatty acid-related genes have been characterized in wax synthesis in model plant and Kentucky bluegrass [14, 20]. Combined with the transcriptome data of overexpression *PpGS1.1* lines, differential changes in fatty acid-related pathways might be the reason for alterations in wax synthesis (Fig. 8c). The expression of *KCS*, *HCD*, and *ACOT* genes involved in the fatty acid elongation pathway were inhibited in transgenic plants (Fig. 8c), which agreed with the idea that fatty acid-related genes participated in pathogens resistance in *Arabidopsis* and *B. napus* accession*s* [18, 19]. Studies also showed that a thinner and more permeable cuticle did not help the entry of these fungi but rather arrested their invasion [13, 14]. In barley, the removal of cuticular waxes reduced conidial germination of *B. graminis* [15]. In this study, overexpression lines favored the reduction of wax content, which was consistent with PM invasion in *Arabidopsis* [40]. Our results suggested that the overexpression of *PpGS1.1* mainly promoted N metabolism and inhibited fatty acid metabolism, which affected the synthesis and antifungal properties of epidermal wax in Kentucky bluegrass [22].

**Figure 9:**
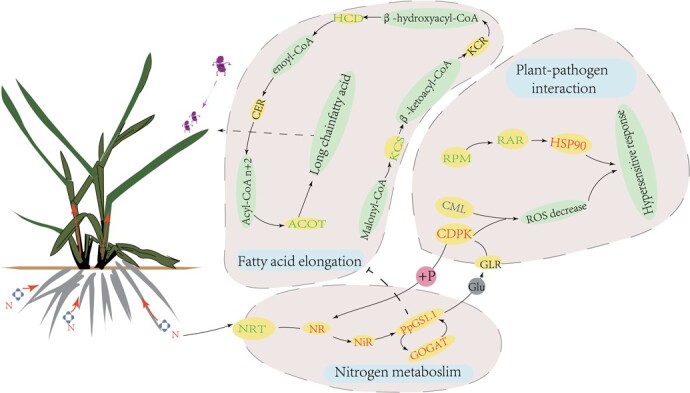
Expression model of *PpGS1.1* gene negatively regulates powdery mildew resistance in Kentucky bluegrass. Blue ellipse background represents the different pathways; green ellipse background represents the nodal matter in the pathways; yellow ellipse background represents the differentially expressed genes. Red, green, and purple fonts represent genes that are upregulated, downregulated, and not significant, respectively.

Collectively, we proposed a hypothetical model for interpreting Kentucky bluegrass defense mechanisms against PM in response to N supplies (Fig. 9). It appeared that ‘N metabolism’ and ‘plant–pathogen interaction’ pathways were closely related, and *PpGS1.1* played a key role in linking the two pathways. The *GS* complex with *CDPK* and *NR* instead of *AS* or *GDH* might respond to PM under HN. Overexpression of *PpGS1.1* increased carbon and N contents and disease severity and inhibited fatty acid metabolism. Our work demonstrated that the *PpGS1.1* gene had a negative regulation of PM resistance in Kentucky bluegrass.

## Materials and methods

### Plant materials, nitrogen treatments, and disease infection

‘Bluemoon’ and ‘Balin’ of Kentucky bluegrass cultivars were used in this experiment. The sods were collected from the field of Northeast Agricultural University (Harbin, China), and then planted in PVC tubes filled with sand and vermiculite (2:1). Grasses were grown in a growth chamber under temperatures of 25/20°C (day/night), a relative humidity of 65%, and light intensity of 700 μmol·m^−2^·s^−1^ for 12 hours. The plants were irrigated every 2 d, cut weekly to about 10.0 cm, and supplied once a week with a 100-mL full-strength Hoagland solution. ‘Bluemoon’ maintained a better turf quality under low N than ‘Balin’ [32]. Grasses were exposed to ten gradient N treatments ranging from 0.5 to 33 mM (Fig S1b and c, see online supplementary material), and three N levels were finally chosen for N treatments in Hoagland solution consisting of 0.5 mM for low N (LN), 15 mM for normal N (NN) and 30 mM for high N (HN) treatments. The HN treatment was used for inducing powdery mildew. Plants received 100 mL of three different N solutions every other day.

Powdery mildew disease was visually rated on a scale of 0 to 9, with 0 indicating no disease spot, 1–3 being minor infection, 4–6 being moderate infection, and 7–9 being severe infection in Kentucky bluegrass (Table S1, see online supplementary material). Powdery mildew infection degree of 38 ecotypes of Kentucky bluegrass was individually screened, and none of them showed complete disease resistance (Tables S1 and S2, see online supplementary material). ‘Balin’ was more susceptible to PM than ‘Bluemoon’ after high N treatment (Fig S1a, see online supplementary material). Plants were grown in different N solutions for one day prior to inoculation with *B. graminis*. The individual isolate was purified by powdery mildew single-colony inoculation on healthy seedlings for five consecutive generations. Different paintbrushes were used to dust purified conidia from one PM patch onto leaves of two cultivars. DNA sequences of conidia (GeneBank ID: MZ452631.1) from the infected leaves further validated the results of the purified *B. graminis* after different N treatments. The experiment was a randomized complete block design with three replications for treatments and cultivars. Leaf sampling was made after 21 d of N treatments for further analyses.

### Morphology, disease index, C and N content

Plant height, length, and width were measured on 10 individual plants of each cultivar. Leaf area was calculated using length and width of the leaf lesion area. The average growth rate of the lesion was measured once every five days. The evaluation criterion of PM occurrence in Kentucky bluegrass after N treatments was the same as mentioned above (Table S1, see online supplementary material).

To measure carbon and N content, leaf tissues were dried at 85°C in an oven, and then ground into powders. A 10 mg aliquot of powder was used for determining carbon and N by a combustion method using the elemental analyser (Vario EL III,ELEMENTAR, Hanau, Germany) [41].

### N-assimilation enzymes

The activity of nitrate reductase (NR) was measured as described previously [42]. Briefly, 0.2 g of fresh leaves were put into 8 mL 0.1 M KNO_3_ solution and vacuumed. After samples were incubated in the dark at 25°C for 30 min, 1 mL of 30% trichloroacetic acid was added to terminate the reaction, followed by adding 1 mL of 0.02% α-naphthylamine and 1% sulfonamide. The absorbance was read at 520 nm. For determining nitrite reductase (NiR) activity, 0.2 g of fresh leaves were homogenized with 5 mL of 50 mM buffer (5 mM EDTA, 5 mM MgCl_2_ and 40 mM phosphate buffer, pH 7.5) and centrifuged at 4°C for 15 min. The supernatant was collected and the absorbance was read at 530 nm [43]. For extraction of crude enzymes for measuring activities of glutamine synthetase (GS) and glutamate synthase (GOGAT), 0.2 g of fresh leaves were homogenized in 5 mL 0.05 M Tris HCl (pH 8.0) on ice, incubated at 4°C for 30 min and centrifuged at 12 000 g for 20 min. For GS activity, the assay mixture included 0.7 mL of crude enzyme extract, 0.1 M Tris–HCl and 80 mM hydroxylamine hydrochloride. A 0.7 mL of 40 mM ATP was then added to the mixture, incubated for 15 min, and centrifuged at 5000 g for 10 min. The absorbance was read at 540 nm [44]. The assay mixture for GOGAT contained 0.3 mL of crude enzyme extract, 1.6 mL 50 mM phosphate buffer (pH = 7.5), 0.4 mL of 50 mM L-glutamine, 0.5 mL of 50 mM a-ketoglutarate, and 0.2 mLof 0.6 mM NADH. The absorbance was read every 30 s at 340 nm for 10 consecutive times. One unit GOGAT activity was calculated by reducing one μmol NADH per min [45].

### *Malondialdehyde (MDA), superoxide* (*O_2_^•-^), and antioxidant determination*

For MDA assay, 0.2 g of leaves were homogenized with 10% of trichloroacetic acid and then centrifuged at 4000 g for 10 min. A 1 mL of supernatant was mixed with 5 mL of 20% trichloroacetic acid with 0.6% thiobarbituric acid. The mixture was heated for 15 min, cooled in ice, and centrifuged at 4000 g for 10 min, and the absorbance was read at 450, 532, and 600 nm [46].

For antioxidant assay, 0.5 g of leaves were homogenized with 2 mL of 50 mM phosphate buffer (pH 7.8). The supernatant was collected after centrifuging at 4°C for 15 min and used for assay of superoxide anion (O_2_^•-^) and enzyme activity. For determining the content of superoxide anion (O_2_^•-^), the mixture contained 1 mL of the extract and 1 mL of 10 mM hydroxylamine hydrochloride. After incubating at 25°C for 20 min, the absorbance was read at 530 nm. The reaction mixture for catalase (CAT) activity contained 0.2 mL of supernatant, 1.5 mL of phosphate buffer (PH 7.8), 1 mL of distilled H_2_O and 0.3 mL of 100 mM H_2_O_2_. The decline in absorbance at 240 nm was recorded for calculating CAT activity [47]. The reaction mixture for measuring POD activity contained 0.1 mL of enzyme extract, 100 mL of 50 mM PBS (pH 6.0), 56 μL of guaiacol, and 38 μL of 30% H_2_O_2_. The increase in absorbance at 470 nm was read and used for calculating POD activity [48]. Activity of SOD was determined according to the method of Havir and McHale (1989) [49]. The SOD reaction mixtures contained 0.1 mL of supernatant, 0.3 mL of 50 mM Na-carbonate (pH 10.2), 0.3 mL of 1.3 mM riboflavin, 0.3 mL of 13 mM methionine, and 0.3 mL of 75 mM NBT. The absorbance was read at 560 nm. One unit of SOD activity was defined as the amount of enzyme inhibiting 50% of nitro blue tetrazolium (NBT) reduction.

### RNA extraction and transcriptome analysis

Leaf RNA was extracted using TRIzol reagent (Invitrogen, Carlsbad, CA, USA), and RNA sequencing was performed in Novogene (Tianjin, China). A total of 18 libraries (2 cultivars × 3 treatments × 3 biological replicates) were sequenced on the Illumina platform (HiSeq 6000), which produced paired-end reads of 150-nucleotide. The adapter sequences and low-quality bases were removed, and the clean data was *de novo* assembled using the Trinity platform (Trinity) [50].

The Expectation Maximization (RSEM) was used to map the transcripts to reference unigenes via RNA-seq. The read count numbers were calculated and translated to FPKM (fragments per kilobase of transcript per million mapped reads) gene values [51]. DESeq was used for analysing differentially expressed genes (DEGs) [52]. We used FDR ≤ 0.05 and |log2Fold > Change| ≥ 1 as threshold for screening DEGs. All identified DEGs were mapped to gene ontology (GO) and Tokyo Encyclopedia of Genes and Genomes (KEGG) databases. The significantly enriched biochemical pathways were obtained using KOBAS with corrected *P*-value ≤0.05.

### Weighted gene co-expression network analysis (WGCNA)

The co-expression modules were constructed using WGCNA in the R package [53], based on M-values (TMM) normalized FPKM values. The adjacency matrix was further converted to a topological overlap matrix (TOM) for identifying modules associated with treatments in the reconstruction network. The transcripts with similar expression patterns were categorized into one module, and hub genes in these modules were subsequently calculated. Finally, the co-expression networks were visualized using Cytoscape software (v.3.5.0) [54].

### PpGS1.1 bioinformatics analysis and genetic transformation

The conserved domain in PpGS1.1 was identified using SMART [55]. The sequence alignments were performed using the Clustal X method. A phylogenetic analysis was constructed using the Neighbor-Joining method with 1000 bootstrap replicates in MEGA 7 (Molecular Evolutionary Genetics Analysis).

RNA was extracted from ‘Bluemoon’ leaves and reverse transcription was performed to obtain cDNA. Using transcriptomics data and RT-PCR methods, the coding sequence (CDS) of *GS1.1* (*PpGS1.1*) gene in *P. pratensis* was obtained and uploaded to the NCBI (GeneBank ID: KY678612.1). The recombinant vector including *PpGS1.1* was constructed under the control of the CaMV 35S promoter. The *35S::PpGS1.1* plasmid was transferred into *Agrobacterium tumefaciens* LBA4404. The primers used in this study are listed in Table S3, see online supplementary material.

‘Bluemoon’ seeds were rinsed five times in sterile deionized water, soaked in 1% sodium hypochlorite solution for 15 min and then placed on MS_1_ medium for 2–3 weeks. The embryogenic callus was placed in resuspending *A. tumefaciens* solution, vacuum-treated for 3 min, and shaken at 100 rpm for 30 min. All infected callus was placed on filter paper to remove the excess liquid, and then transferred into the subculture medium, and co-cultured for 3 days in the dark at 25°C. The co-cultured calluses were washed with sterile water and 200 mg·L^−1^ amoxicillin aqueous solution. MS_2_ differentiation medium was then used to select and cultivate callus. The differentiation stage was treated in the dark at 25°C for 6 weeks and the selection medium was changed every 2 weeks. To culture resistant seedlings, the differentiated shoots were transferred to regeneration medium without antibiotics. The mediums used in this study are listed Table S4, see online supplementary material.

PCR and qPCR experiments were performed for identification of the successfully transformed lines. DNA and RNA of both wild-type and transformant lines were isolated by using the method described by de Kochko and Chang [56, 57]. The primers used in PCR reactions are shown in Table S3, see online supplementary material.

### Scanning electron microscope (SEM) observation

Fresh leaves were cut into small squares with 5 mm side length around veins, immediately placed in vials containing 2.5% glutaraldehyde for 2 h, and fixed with 0.1 mM PBS (pH 7.2) three times with 10 min each time. The leaves were gradually dehydrated for 15 min in 30%, 50%, 60%, and 70% ethanol solutions, respectively. Leaves were then transferred to tert-butanol (pure) solution for 20 min. Finally, the vial containing tert-butanol solution was placed in an icebox for 15 min and then the samples were dried in a freeze dryer. After ice crystals were evaporated and dried in the vial, the samples were spattered with gold-plated film in an ion coater, observed and imaged by SEM (Hitachi SU-8010, Tokyo, Japan) [58].

### Subcellular localization of *PpGS1.1*

To construct the GFP fusion genes, the ORF of *PpGS1* without the stop codon was amplified by PCR and cloned to the *XbaI* and *KpnI* sites of the pYBA1132-GFP vector. The ligated product was transformed into DH5α competent cells. The expression vector assembly by colony PCR primers was verified (Table S3, see online supplementary material) and positive clones were chosen for sequencing. The pYBA1132-*PpGS1.1*-GFP vector and nuclear marker gene *OsGHD7* were co-transformed into rice protoplasts for transient expression. After 48 hours of dark culture, the expression of *PpGS1.1* in rice protoplasts was observed under a laser copolymerization microscope [59].

### RNA-seq analysis of PpGS1.1-overexpression lines (OE) and wild type (WT)

To identify transcripts for PM resistance in Kentucky bluegrass, we performed transcriptomic analyses of the leaf under natural growth conditions. Total RNA was extracted from the leaf veins of WT and OE lines, as described previously [55]. cDNA libraries were then constructed and sequenced using Illumina platform (HiSeq 6000), and raw reads were cleaned and filtered. Gene expression levels were then estimated with FPKM [50]. DESeq was used to detect DEGs between the OE and WT lines with the following criteria: *P*-value ≤0.05 and |log2Fold > Change| ≥ 1. All DEGs were mapped to the GO and KEGG databases.

### Data analysis

The experimental data were analysed using one-way ANOVA by using Statistical Product and Service Solutions (SPSS) (SPSS Inc., Chicago, IL, USA). The significance was determined using a *t*-test with *P* < 0.05. Analysis of correlation of individual growth parameters was performed using SPSS. Figures were made using R (www.r-project.org) and GraphPad Prism v.9.00 (Graphpad Company, USA). The heatmaps were drawn via TBtools software [60].

## Acknowledgements

This research was supported by National Natural Science Foundation of China (Grant No. 31971772; 31772354; 32001407).

## Author contributions

X.S. performed the experiment, analysed data and wrote the manuscript; Z.G., Z.S., L.Q., L.D., L.X., R.Y., and W.D. assisted in data collection; F.X. and Y.C. designed and supervised the experiment; Y.J. contributed to data interpretation and manuscript writing. All authors approved the publication of the manuscript.

## Data availability

Data supporting the conclusions can be obtained in the publication and its supplemental materials. Any additional relevant information may be found from the corresponding authors (F.X. and Y.C.).

## Conflict of interests

The authors declare that they have no conflicts of interest.

## Supplementary data

Supplementary data is available at *Horticulture Research* online.

## Supplementary Material

supp_data_uhac196
