# Supplementary material for: Glutamine synthetase gene PpGS1.1 negatively regulates the powdery mildew resistance in Kentucky bluegrass
Source: Hortic Res. 2022 Aug 30;9:uhac196. doi: 10.1093/hr/uhac196 (PMC9677456; doi:10.1093/hr/uhac196)
Supplement: supp_data_uhac196 [file supp_data_uhac196.zip › Supplemental.docx]

| **Supplementary Table S1. Classification criteria of powdery mildew disease in Kentucky bluegrass** | |
| --- | --- |
| Disease level | Disease symptoms |
| 0 | Without disease spot |
| 1-3 | Minor infection, 5%-10% spot of the leaves |
| 4-6 | Moderate infection, 11%-30% spot of the leaves |
| 7-9 | Severe infection, above 30% spot of the leaves |

| **Supplementary Table S2. Nitrogen content and disease degree of powdery mildew in 38 Kentucky bluegrass ecotypes** | | | | | |
| --- | --- | --- | --- | --- | --- |
| Name | N concent (%) | Disease degree | Name | N concent (%) | Disease degree |
| Sn011 | 4.6 ± 0.02 | 0.5 | Gzz056 | 1.9 ± 0.14 | 9.0 |
| Balin | 3.2 ± 0.19 | 1.0 | GreenEmpire | 1.9 ± 0.10 | 1.0 |
| Hy312 | 2.9 ± 0.22 | 0.5 | Xh003 | 1.9 ± 0.11 | 2.0 |
| Blueberry | 2.6 ± 0.38 | 2.0 | Euromyth | 1.9 ± 0.04 | 4.0 |
| Marauder | 2.6 ± 0.06 | 0.5 | Award | 1.9 ± 0.10 | 0.5 |
| Merit | 2.4 ± 0.11 | 6.0 | Supermerit | 1.8 ± 0.11 | 0.5 |
| Moonlight | 2.4 ± 0.18 | 3.5 | Brootlawn | 1.8 ± 0.11 | 2.0 |
| Touchdown | 2.4 ± 0.13 | 0.5 | Eclipse | 1.7 ± 0.19 | 1.0 |
| Best | 2.3 ± 0.16 | 0.5 | Qb006 | 1.7 ± 0.05 | 0.5 |
| Kentucky | 2.3 ± 0.24 | 9.0 | America | 1.7 ± 0.10 | 0.5 |
| Midnight | 2.2 ± 0.21 | 8.5 | Brilliant | 1.7 ± 0.05 | 0.5 |
| Bluechip | 2.2 ± 0.29 | 0.5 | Nassau | 1.7 ± 0.09 | 5.0 |
| Xal005 | 2.2 ± 0.18 | 0.5 | Baron | 1.6 ± 0.12 | 0.5 |
| Total Eclipse | 2.2 ± 0.06 | 8.0 | Thermal Blue | 1.6 ± 0.08 | 8.0 |
| Everglade | 2.2 ± 0.12 | 8.5 | Washington | 1.6 ± 0.13 | 0.5 |
| Highnoon | 2.1 ± 0.17 | 1.0 | Nw002 | 1.6 ± 0.03 | 0.5 |
| NublePlus | 2.0 ± 0.07 | 6.0 | Alpine | 1.5 ± 0.07 | 0.5 |
| Impact | 2.0 ± 0.09 | 8.0 | Evergreen | 1.5 ± 0.13 | 0.5 |
| Challenger | 1.9 ± 0.18 | 0.5 | Bluemoon | 1.4 ± 0.15 | 1.0 |

| **Supplementary Table S3. PCR primers used in this study** | |
| --- | --- |
| Primer name | Sequence (5’-3’) |
| PpGS1.1 F | GCTCTAGATCAGGGCTTCCACAGGAGGG |
| PpGS1.1 R | GGGGTACCATGGCGCTCCTCACCGATCT |
| Hpt F | ACACTACATGGCGTGATTTCAT |
| Hpt R | TCCACTATCGGCGA-GTACTTCT |
| PpGSGFP F | CTGCAGATGGCGCAGGCGGTGGTGC |
| PpGSGFP R | GAATTCTACATTAATCGACAGCTTC |

| **Supplementary Table S4 Medium used in this study** | | |
| --- | --- | --- |
| Medium name | Composition | PH |
| MS_1_ | MS+0.1 mg·L^-1^ 6BA+3 mg·L^-1^ 2,4-D+8% agar | 5.8 |
| MS_2_ | MS+3 mg·L^-1^ 6BA+0.5 mg·L^-1^ NAA+8% agar | 5.8 |

| **Supplementary Table S5 Correlation analysis between N assimilation enzyme activities and antioxidant metabolism across two cultivars and three N levels** | | | | | | | | | |
| --- | --- | --- | --- | --- | --- | --- | --- | --- | --- |
| Treatments | NR | NiR | GS | GOGAT | O_2_^•-^ | MDA | SOD | CAT | POD |
| NR | 1 | 0.87** | 0.88** | 0.77** | 0.43* | -0.37 | 0.77** | 0.85** | 0.96** |
| NiR |  | 1 | 0.90** | 0.90** | 0.65** | -0.19 | 0.65** | 0.93** | 0.84** |
| GS |  |  | 1 | 0.92** | 0.59** | -0.21 | 0.75** | 0.81** | 0.90** |
| GOGAT |  |  |  | 1 | 0.71** | 0.04 | 0.56** | 0.76** | 0.73** |
| O_2_^•-^ |  |  |  |  | 1 | 0.45* | 0.02 | 0.48* | 0.35 |
| MDA |  |  |  |  |  | 1 | 0.72** | -0.31 | -0.47* |
| SOD |  |  |  |  |  |  | 1 | 0.68** | 0.85** |
| CAT |  |  |  |  |  |  |  | 1 | 0.82** |
| POD |  |  |  |  |  |  |  |  | 1 |

*, ** indicate significance at *P* < 0.05, and 0.01, respectively.

| **Supplementary Table S6. The top 20 enriched pathway terms in the GO database** | | | |
| --- | --- | --- | --- |
| GO_accession | Description | Term_type | pValue |
| GO:0043531 | ADP binding | molecular_function | 9.34E-14 |
| GO:0055114 | oxidation-reduction process | biological_process | 3.65E-12 |
| GO:0016491 | oxidoreductase activity | molecular_function | 1.55E-11 |
| GO:0003824 | catalytic activity | molecular_function | 2.00E-11 |
| GO:0008152 | metabolic process | biological_process | 9.99E-10 |
| GO:0032559 | adenyl ribonucleotide binding | molecular_function | 1.11E-09 |
| GO:0030554 | adenyl nucleotide binding | molecular_function | 1.20E-09 |
| GO:0000166 | nucleotide binding | molecular_function | 1.32E-09 |
| GO:1901265 | nucleoside phosphate binding | molecular_function | 1.32E-09 |
| GO:0036094 | small molecule binding | molecular_function | 2.60E-09 |
| GO:0044710 | single-organism metabolic process | biological_process | 2.83E-09 |
| GO:0001883 | purine nucleoside binding | molecular_function | 5.24E-09 |
| GO:0032549 | ribonucleoside binding | molecular_function | 5.24E-09 |
| GO:0032550 | purine ribonucleoside binding | molecular_function | 5.24E-09 |
| GO:0032555 | purine ribonucleotide binding | molecular_function | 5.50E-09 |
| GO:0001882 | nucleoside binding | molecular_function | 5.73E-09 |
| GO:0017076 | purine nucleotide binding | molecular_function | 5.92E-09 |
| GO:0032553 | ribonucleotide binding | molecular_function | 7.61E-09 |
| GO:0097367 | carbohydrate derivative binding | molecular_function | 9.16E-09 |
| GO:0043168 | anion binding | molecular_function | 4.35E-08 |


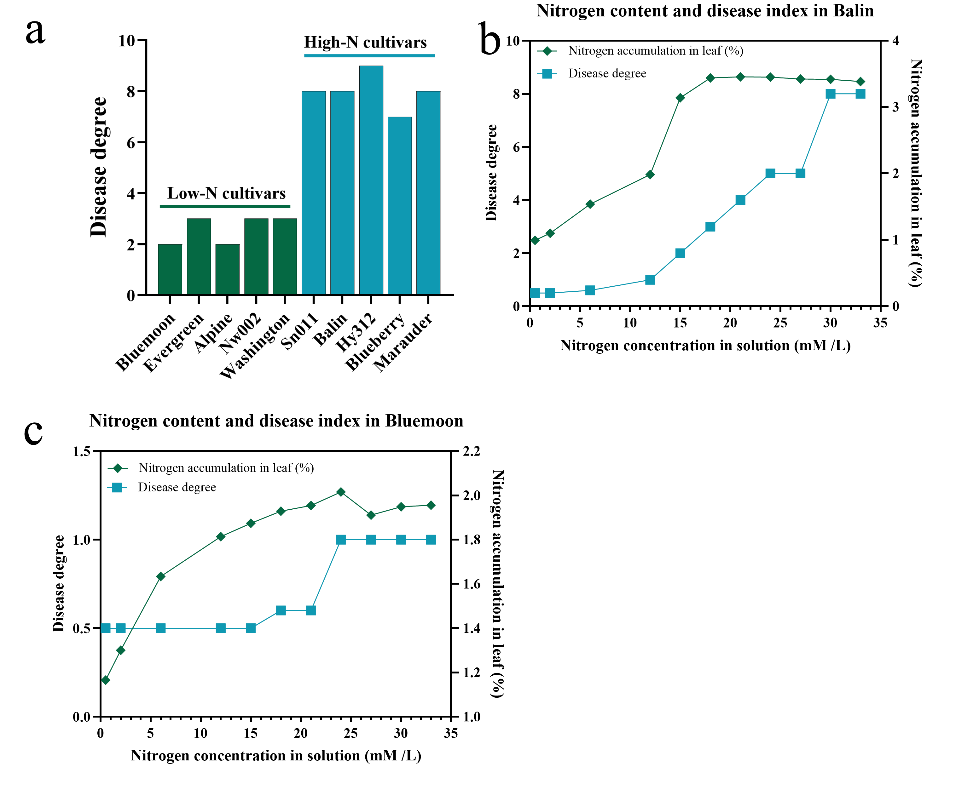


**Supplementary Fig S1. Powdery mildew disease degree evaluation. a**. The powdery mildew disease degree of five high-N cultivars and five low-N cultivars after high N treatment. The green and blue bars represent high-N cultivars and low-N cultivars, respectively. **b, c**. Disease degree and N content in leaf after different N concentration treatments in Balin and Bluemoon, respectively.


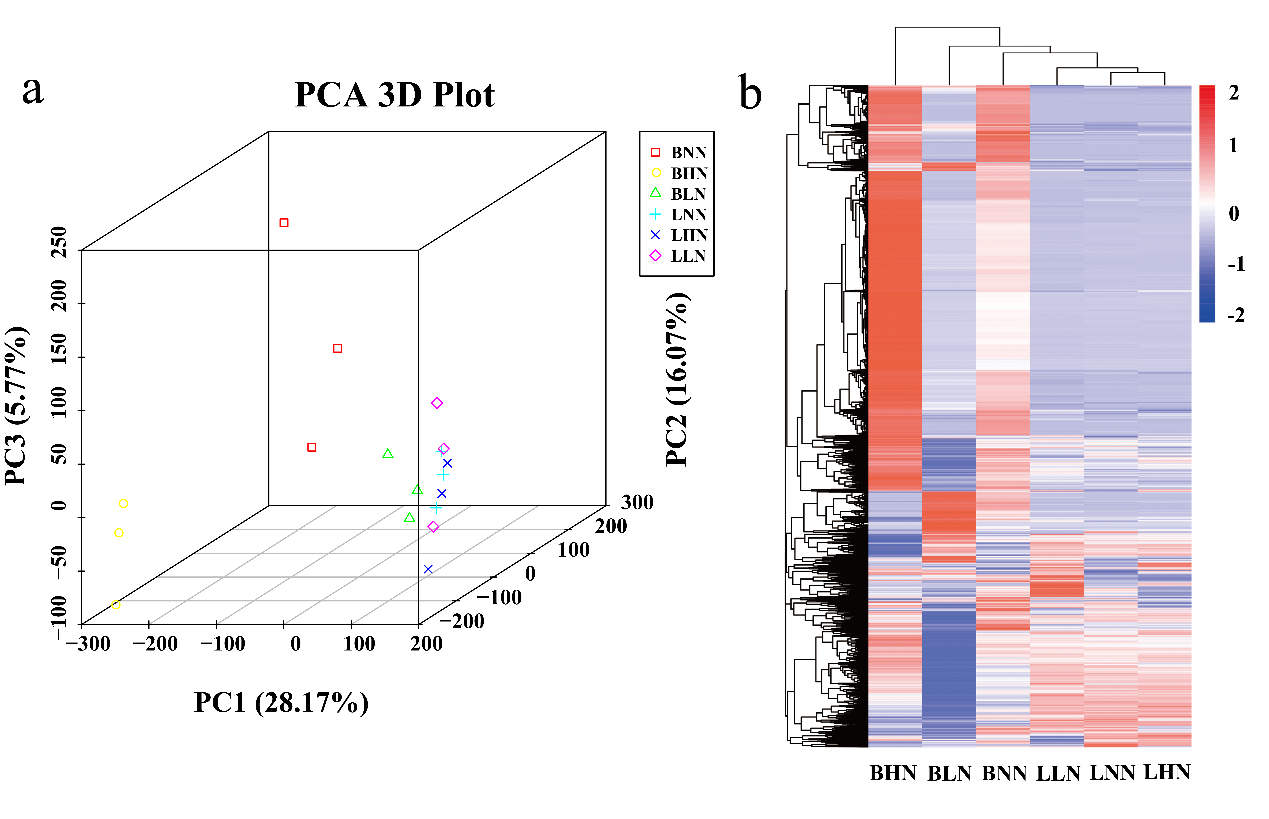


**Supplementary Fig S2. Transcriptome information of Kentucky bluegrass cultivars in response to N treatments. a.** The principal component analysis (PCA) of differently expressed transcripts during N treatments. **b.** Hierarchical clustering analysis of N induced changes in gene expression of Kentucky bluegrass. (BLN, BNN, BHN = Balin under low, normal and high N treatments, respectively. LLN, LNN, LHN = Bluemoon under low, normal and high N treatments, respectively.).


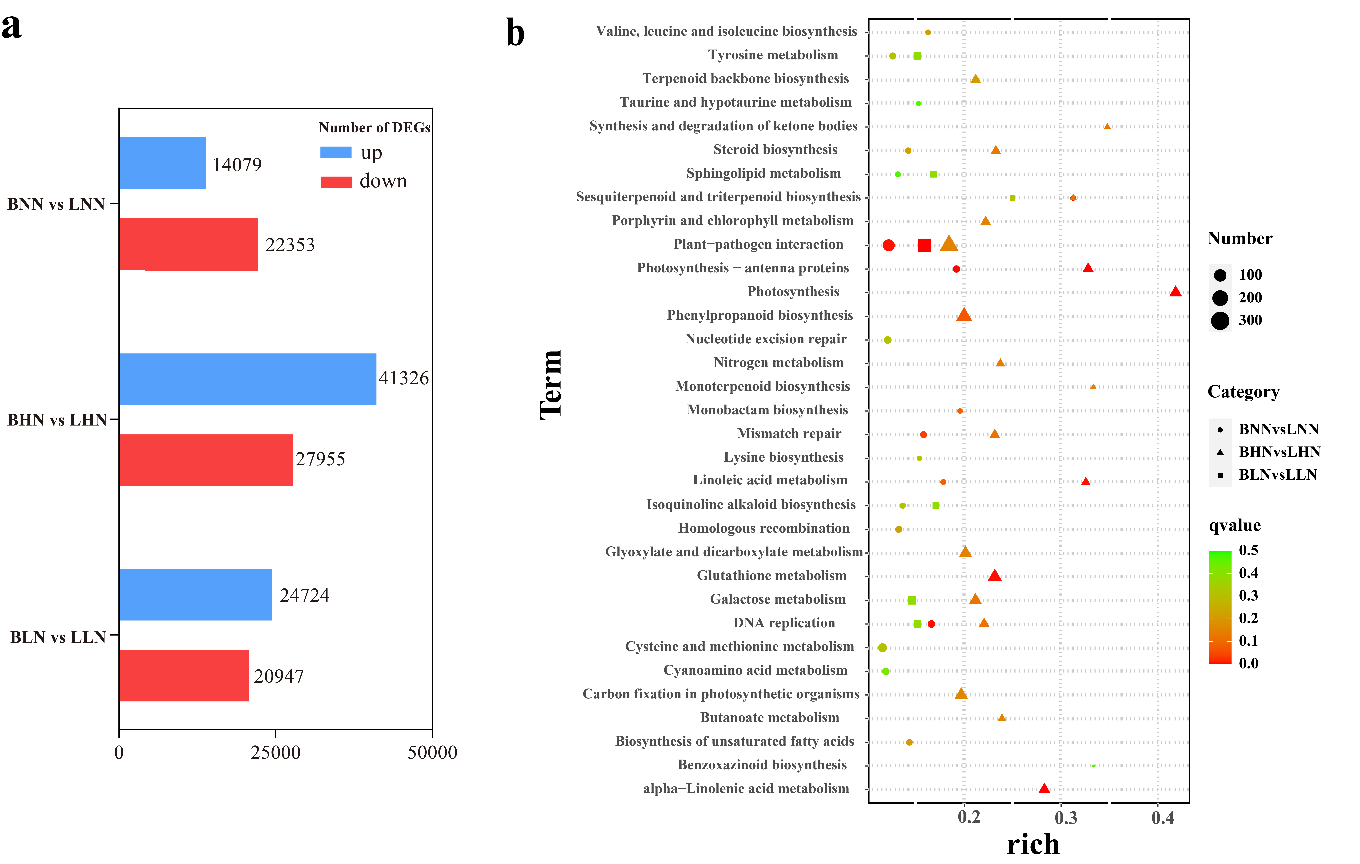


**Supplementary Fig S3. Differentially expressed genes** (**DEGs) statistics and KEGG analysis in Balin and Bluemoon based on RNA-seq under different N treatments. a.** DEGs statistics. Blue and red bars mean upregulated and downregulated genes. **b.** Scatterplot of KEGG pathways enriched for DEGs. The q value is the corrected *P* value and ranges from 0 to 1, and a lower q value indicates greater intensity. The size of the circles indicates the number of genes. (BLN, BNN, BHN = Balin under low, normal and high N treatments, respectively. LLN, LNN, LHN = Bluemoon under low, normal and high N treatments, respectively.).


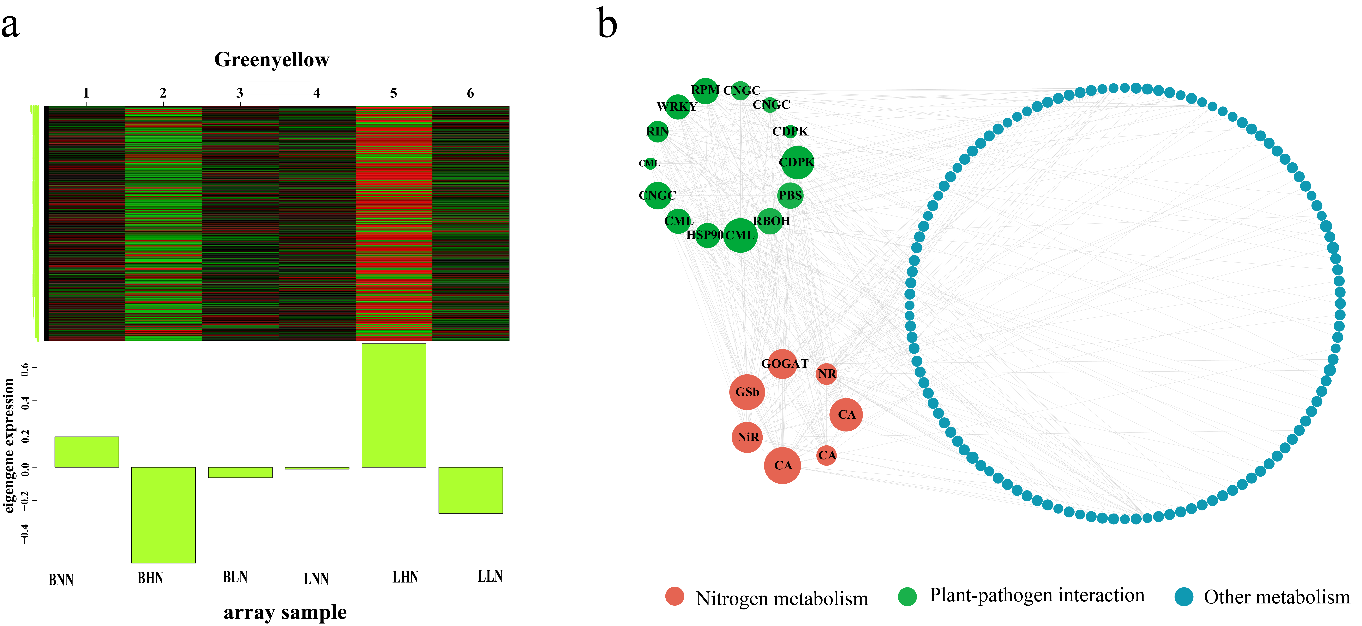


**Supplementary Fig S4. Visual analysis of co-expression correlation of genes in greenyellow module**. **a.** Co-expressed genes in brown module is shown in bar graphs. **b.** Visual analysis of co-expression correlation of genes in brown module via Cytoscape. Each node represents a gene, and the connecting lines (edges) between genes represent co-expression correlation. The size of circles is proportional to connectivity. The red, green and blue circles represent genes in ‘nitrogen metabolism’, ‘plant-pathogen interaction’, and other metabolism pathways, respectively. (BLN, BNN, BHN = ‘Balin’ under low, normal and high nitrogen treatments, respectively. LLN, LNN, LHN = ‘Bluemoon’ under low, normal and high nitrogen treatments, respectively.).


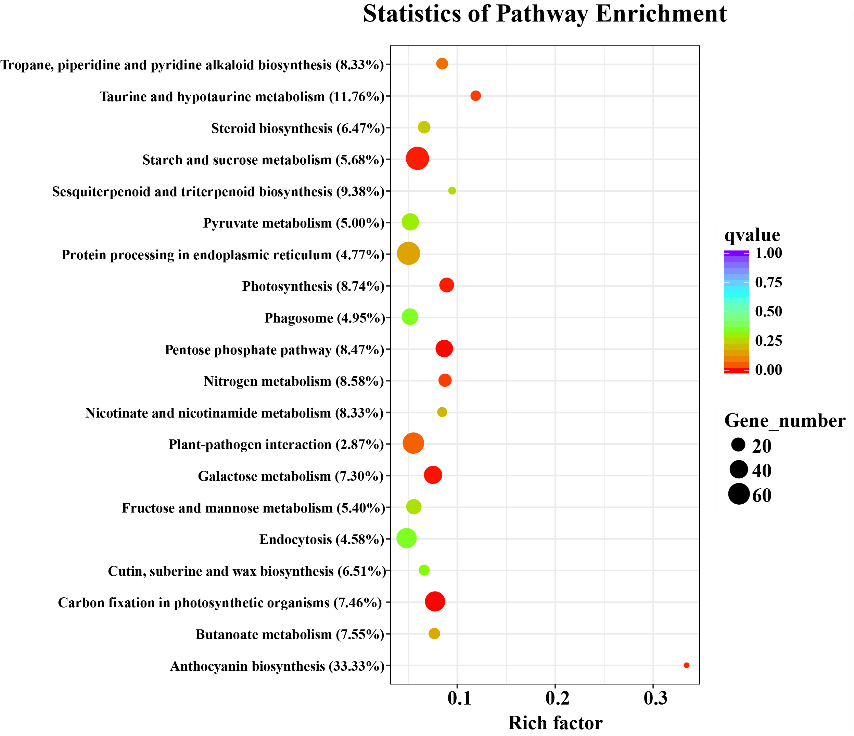


**Supplementary Fig S5. KEGG analysis differentially expressed genes** (**DEGs) in brown module.** Scatterplot of KEGG pathways enriched for DEGs. The q value is the corrected *P* value and ranges from 0 to 1, and a lower q value indicates greater intensity. The size of the circles indicates the number of genes.


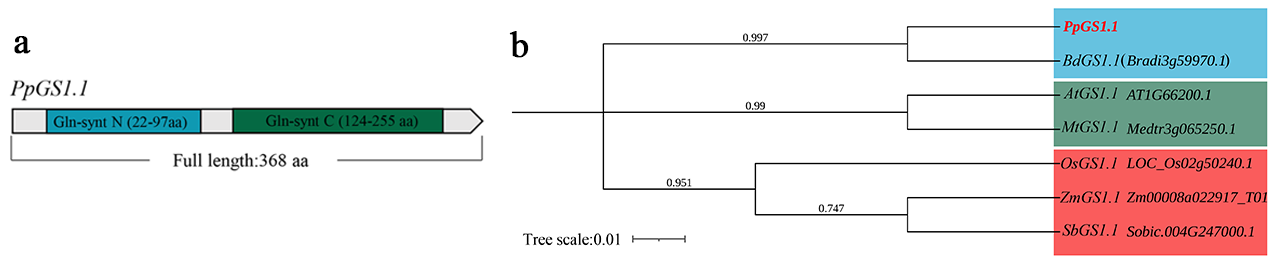


**Supplementary Fig S6. Analysis of protein domain and phylogenetic tree of PpGS1.1.** **a.** Illustrative structure of PpGS1.1; **b.** Neighbor-Joining phylogenetic tree of PpGS1.1 and eight orthologous GS1.1. The N-J tree was constructed using MEGA7. The orthologous proteins were of *Brachypodium distachyon* (BdGS1.1), *Arabidopsis* (AtGS1.1), *Medicago truncatula* (MtGS1.1), rice (OsGS1.1), maize (ZmGS1.1) and sorghum (SbGS1.1).


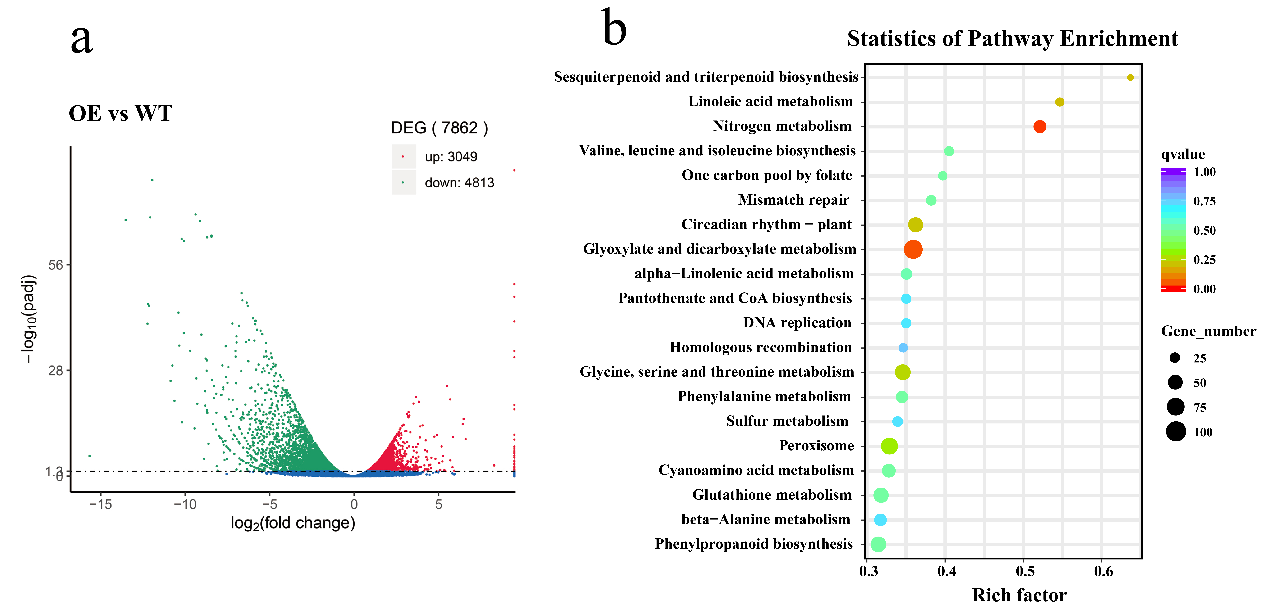


**Supplementary Fig S7. Volcano plots and KEGG analysis DEGs in overexpression lines (OE) and wild type based on transcriptome sequencing. a.** Volcano plots showing the number of differentially expressed genes (DEGs) in OE vs WT. DEGs are shown in red (upregulated) and green (downregulated), while blue indicates genes that were not differentially expressed. **b.** Scatterplot of KEGG pathways enriched for DEGs in OE vs WT. The q value is the corrected *P* value and ranges from 0 to 1, and a lower q value indicates greater intensity. The size of the circles indicates the number of genes. The top 20 enriched pathway terms in the KEGG database are listed.
